# Cross-Species Upregulation of *MAGED2* in Liver Cancer Suggests a Role in Obesity-Driven Tumor Progression

**DOI:** 10.3390/cimb48020139

**Published:** 2026-01-27

**Authors:** Tara Bayat, Farzana Yeasmin Popy, Rebecca R. Florke Gee, Benjamin Barr, Yusuff Olayiwola, Juan Sebastian Solano Gutierrez, Denis Štepihar, Jorge Diaz-Riaño, Stephanie Myers, Kaja Blagotinšek Cokan, Damjana Rozman, Lauren Gollahon, Klementina Fon Tacer

**Affiliations:** 1 School of Veterinary Medicine, Texas Tech University, 7671 Evans Dr., Amarillo, TX 79106, USA; 2 Texas Center for Comparative Cancer Research (TC3R), 7671 Evans Dr., Amarillo, TX 79106, USA; 3 Department of Biological Sciences, Texas Tech University, 2500 Broadway, Lubbock, TX 79106, USA; 4 Department of Kinesiology and Sport Management, Texas Tech University, Lubbock, TX 79106, USA; 5 Vascular Biology Center, Medical College of Georgia, Augusta University, Augusta, GA 30912, USA; 6 Systems and Computing Engineering Department, Universidad de los Andes, Bogotá 111711, Colombia; 7 Center for Functional Genomics and Bio-Chips, Institute of Biochemistry and Molecular Genetics, Faculty of Medicine, University of Ljubljana, Zaloška cesta 4, 1000 Ljubljana, Slovenia; 8 Molecular Biology Laboratory, BIA Separations CRO, Labena Ltd., 1000 Ljubljana, Slovenia

**Keywords:** hepatocellular carcinoma, liver tumors, melanoma-associated antigen (MAGE), obesity, high-fat diet, comparative cancer research, cancer-testis antigen (CTA)

## Abstract

Melanoma-associated antigens (MAGEs) are cancer-testis antigens (CTAs) aberrantly expressed in multiple cancer types, including hepatocellular carcinoma (HCC), and associated with aggressive phenotypes. Although MAGE proteins are widely studied as cancer immunotherapy targets, their roles in HCC and the regulation of their expression during liver pathogenesis in mouse models, including dietary effects, remain poorly understood. We analyzed *Mage* gene expression in liver tissues from 78 C3H/HeJ mice with chronic diet-induced obesity. While type I *MAGE* genes are frequently expressed in human HCC, we found no evidence of their expression in mouse liver tumors, suggesting species-specific regulation. In contrast, type II *Maged2*, previously reported to be upregulated in human HCC, was significantly increased in mouse liver tumors. Analysis of human HCC samples from The Cancer Genome Atlas (TCGA) database confirmed *MAGED2* upregulation and its association with patient prognosis. Together, these findings identify *MAGED2* as a conserved marker of liver cancer in both humans and mice and emphasize the importance of cross-species comparative approaches for selecting appropriate models and accurately interpreting results, particularly for CTAs, which often evolved recently and in a species-specific manner.

## 1. Introduction

Hepatocellular carcinoma (HCC), the most common form of primary liver cancer, exhibits an asymptomatic progression that makes it difficult to effectively diagnose during early stages, contributing to its poor prognosis and high mortality rate [1,2,3,4]. Although the etiology of HCC is not fully understood, epidemiological studies have identified several risk factors, including infection with hepatitis B or C viruses, excessive alcohol consumption, and autoimmune diseases, as well as obesity and its associated comorbidities, such as type II diabetes and metabolic dysfunction-associated steatotic liver disease/metabolic dysfunction-associated steatohepatitis (MASLD/MASH) [4,5,6,7,8]. Therapeutic options for HCC include surgical resection, radiofrequency ablation, liver transplantation, or the more recently developed strategies of targeted therapy and immunotherapy [9,10,11]. Despite significant advancements in treating HCC, overall survival remains poor due to its metastatic propensity and high recurrence rate [1,12]. Thus, a better understanding of the molecular mechanisms underlying HCC development and progression will be key to identifying biomarkers for early diagnosis and prognosis prediction and designing novel therapies to ultimately improve the prognosis of HCC patients.

In recent years, immunotherapy has revolutionized HCC treatments [3]. Immune checkpoint inhibitors targeting the PD1/PDL1 pathway were recently approved for advanced HCC treatment [13]. The best available first-line treatment for patients with advanced HCC is a combination of PDL1 blockade with atezolizumab and VEGF blockade with bevacizumab [4,14,15]. In addition to checkpoint inhibitors, researchers are pursuing other immunotherapy approaches for HCC treatment, including adoptive cell transfer therapy, chimeric antigen receptor T-cell (CAR-T) therapy, tumor-infiltrating lymphocytes, engineered cytokines, and therapeutic cancer vaccines [13,16]. One of the major unmet challenges in HCC immunotherapy is the lack of predictive biomarkers [14]. Recent studies report that a group of genes called melanoma-associated antigens (*MAGEs*) are dysregulated in HCC and associated with aggressive tumor characteristics and poor prognosis [2,12,17,18]. Many members of the *MAGE* family have garnered interest as biomarkers and immunotherapy targets due to their expression in several human cancers, where they can act as tumor antigens and activate an immune response [19,20,21,22,23]. The *MAGE* family consists of approximately 40 genes in humans and mice that are subdivided into type I and type II based on tissue expression and sequence homology [20,24]. Type I *MAGEs*, also known as cancer-testis antigens (CTAs), comprise the *MAGEA*, -*B*, and -*C* genes and are primarily expressed in the testis but are often aberrantly expressed in cancer [25,26]. The *MAGED*, -*E*, -*F*, -*G*, -*H*, -*L*, and *NECDIN* genes make up the type II *MAGEs*, which are more ubiquitously expressed and not typically associated with cancer [20,26]. Some type II genes are implicated in neurodevelopmental diseases, such as *MAGEL2* and *NECDIN* in Prader-Willi and Schaaf-Yang syndromes [27,28]. In human HCC, several *MAGEs* are expressed, but how these genes contribute to HCC development and progression is not yet fully elucidated [2,12,18,29,30,31].

Understanding the expression of *Mages* in mouse models of HCC is a vital part of future basic and translational studies seeking to determine how dysregulated *MAGEs* contribute to HCC prognosis and therapy. In humans, type I MAGEA and -C2 proteins were found to cooperate with their E3 ubiquitin ligase partner TRIM28 to act as oncogenes by ubiquitinating and degrading the central metabolic regulator AMPK [25,32]. In HCC, MAGE–TRIM28-mediated proteasomal degradation was shown to target the gluconeogenic enzyme FBP1, providing a key mechanism for FBP1 downregulation in HCC [33]. These findings reveal that MAGE–TRIM28–driven reprogramming of cancer cell metabolism and HCC tumorigenesis is mediated, at least in part, through FBP1 degradation and subsequent metabolic rewiring [33]. We previously found that in mice, type I *Mages* protect germ cells from various stressors, including nutrient stress, and that this metabolic adaptation can be co-opted by human cancer cells [26,34]. However, it remains unclear whether diet or metabolic status affects the activation or regulation of *MAGE* genes across species and during tumorigenesis.

Here, we aimed to determine the expression of *Mages* in liver tumors that developed in mice fed high-fat diets containing different protein sources and ammonium hydroxide enhancement (AHE). We hypothesized that *MAGEs* are aberrantly expressed in tumors induced by a high-fat diet and that the expression of type I and type II *MAGEs* differs in a species-specific manner between human and murine HCC. Although type I *MAGEs* are frequently activated in human HCC [2,12,17,18], we found that these genes were not expressed in murine liver tumors. Rather, we observed elevated levels of *Maged2* in mouse liver tumors. Then, we analyzed expression data from the The Cancer Genome Atlas (TCGA) database and confirmed the upregulation of *MAGED2* in human HCC samples. These results suggest that *MAGED2* may contribute to liver cancer development or progression in both humans and mice, warranting future investigation into *MAGED2* as a potential diagnostic or therapeutic target.

## 2. Materials and Methods

### 2.1. Animal Study and Diets

Selection of the C3H/HeJ mouse strain was based on disease susceptibility, lifespan, and less aggressive social behavior [35]. Mouse handling followed regulatory compliance under the approved Texas Tech University IACUC protocol number 19021-02. For this study, C3H/HeJ mice were obtained from Jackson Laboratories (Bar Harbor, ME, USA) at 4 weeks old. After one-week acclimatization, mice were randomly separated by cage into the following diet groups: control chow alone (CC) or with AHE (CCN; 380 kcal/100 g, 11% energy as fat), high-fat casein alone (HFC) or with AHE (HFCN; 460 kcal/100 g, 46% energy as fat), high-fat beef protein alone (HFB) or with AHE (HFBN; 470 kcal/100 g, 46% energy as fat). At the initiation of the study, each diet group included 32 male and 32 female mice. As part of a previously published study, terminal cross-sectional tissue collections (censoring) were performed every 6 months on 8 male and 8 female mice, as available, from each diet group [36]. All diets were formulated by Research Diets, Inc. (New Brunswick, NJ, USA), and dietary components were identical to the previous study [36]. All personnel involved were aware of group allocations. Females and males were housed in separate rooms, four mice per GM500 cage (Tecniplast, West Chester, PA, USA), at 22 °C, 30–40% humidity, in 12 h light/dark cycle-controlled rooms. Animals had ad libitum access to water and their assigned diet in pellet form. Mice were maintained on their respective diets for 18 months, at which point the study was terminated because several groups were no longer viable. Mice were euthanized after a 1 h fast using CO_2_ followed by cervical dislocation. Survivability curves and complete liver tumor counts for all diets, except the CCN group, were previously published [36,37].

Hepatocyte-specific *Cyp51* knockout (LKO) mice [38], which begin developing liver tumors at 12 months of age, were generated and used in experiments previously approved by the Veterinary Administration of the Republic of Slovenia (license numbers 34401-31/2011/4 and 34401-52/2012/3) [39]. Animals were housed at 22 °C with 55 ± 10% humidity and maintained on standard rodent chow (diet 1324, Altromin, Lage, Germany) and acidified tap water (pH 3) ad libitum under a 12 h light/dark cycle in groups of 3–5 per cage. At 1, 1.5, and 2 years of age, mice were euthanized by cervical dislocation between 11 a.m. and 1 p.m. following a 4–6 h fast. Liver tissues were collected, with portions fixed in 4% formalin and embedded in paraffin for histological analysis. Additional sections were snap-frozen in liquid nitrogen and stored at −80 °C for subsequent analyses. Except for one male wild-type (WT) mouse, only LKO mice developed tumors [39]. For reverse transcription quantitative PCR (RT-qPCR) experiments, RNA was isolated from normal liver tissue of WT mice and from tumor tissue of LKO mice. In addition, previously published microarray data from *Cyp51* LKO mice were analyzed to complement and validate our findings (GSE58271 and GSE127772) [38,39].

### 2.2. Histology

Immediately after euthanasia, liver and tumor tissues were collected. Liver tumors were identified based on macroscopically visible overgrowth; some lesions were histologically examined and showed compression of normal tissue or distortion of lobular architecture. Portions of normal liver and tumor tissues were fixed in 10% formalin (cat. #BP531-500, Fisher Scientific, Waltham, MA, USA), embedded in paraffin using a Leica EG1160 tissue embedding station (Leica Microsystems, Wetzlar, Germany) and sectioned at 5 µm. Sections were stained with Mayer’s hematoxylin (cat. #AB245882, Abcam, Cambridge, United Kingdom) and eosin Y (Surgipath cat. #3801600, Leica Biosystems, Deer Park, IL, USA) as previously described [36], and histopathologic examinations were performed by an experienced board-certified veterinary anatomic pathologist (Table S1). The tumors were identified histologically by abnormal tissue density, cell shape, and atypical nuclei [36,37].

### 2.3. RNA Preparation

Liver tissues collected from mice after euthanasia were snap-frozen in liquid nitrogen. Approximately 5 mm^3^ of tissue was pulverized with a Spectrum Bessman tissue pulverizer (Fisher Scientific, Waltham, MA, USA) and cryogenically homogenized using the PowerGen 125 Homogenizer (Fisher Scientific, Waltham, MA, USA). Total RNA was extracted with TRIzol reagent (cat. #15596026, Invitrogen, Carlsbad, CA, USA) or the RNeasy Mini Kit (Qiagen, Hilden, Germany) following the manufacturer’s protocols. RNA yield and purity were assessed using a NanoDrop 2000 spectrophotometer (Thermo Scientific, Waltham, MA, USA) or a BioTek Take3 Micro-Volume Plate on a Cytation 5 reader (Agilent, Santa Clara, CA, USA).

### 2.4. Reverse Transcription Quantitative PCR (RT-qPCR) Analysis

Gene expression levels were measured by RT-qPCR as previously described [26]. Briefly, 4 µg of total RNA was treated with DNase I (Roche, Basel, Switzerland or Qiagen, Hilden, Germany) to remove genomic DNA and reverse-transcribed into cDNA using the High-Capacity cDNA Reverse Transcription Kit (Applied Biosystems, Waltham, MA, USA). qPCR was performed in triplicate using 10 ng of cDNA per well in a 384-well plate on a QuantStudio 7 Pro Real-Time PCR System (Applied Biosystems, Waltham, MA, USA) with PowerUp SYBR Green Master Mix (cat. #A25742, Applied Biosystems, Waltham, MA, USA). 18S ribosomal RNA was used as the reference gene, and RT control reactions confirmed that amplification originated from RNA [26]. Primers were previously designed and validated as described [40]. Briefly, they were designed using the Roche Universal Probe Library Assay Design Center and used at a final concentration of 150 nM (sequences listed in Table S3 of [26]). Standard cDNA from mouse testis RNA was used for primer validation and efficiency calculations, determined from standard curve slopes. RT-qPCR data were analyzed using QuantStudio Design and Analysis 2 (DA2) software (Thermo Fisher Scientific, Waltham, MA, USA) and Microsoft Excel. Baselines were set automatically, and thresholds were kept constant to obtain normalized cycle times. Normalized mRNA levels are expressed as arbitrary units (AU) using a modified ΔΔCt method, where efficiency-corrected target values were divided by 18S values and multiplied by 1 × 10^5^ for scaling. Expression was considered absent when Ct > 34 and the difference from the −RT control was ≤2 Ct. Analyses were performed on RNA extracted and reverse-transcribed in two independent preparations, and all genes—including type I and II *Mages*, *Trim28*, *Fgf21*, and *18S* rRNA—were assayed under identical conditions.

### 2.5. RNA Sequencing (RNA-Seq) and Data Analysis

RNA was isolated from normal livers and liver tumors of 18-month-old female and male C3H/HeJ mice for RNA-seq, totaling 60 samples. Random liver sections (~30 mg) were flash-frozen in liquid nitrogen and cryogenically homogenized for 3 min at 2000 rpm using a Mini-BeadBeater-96 (BioSpec Products, Bartlesville, OK, USA) with 2 mm beads. RNA was extracted with the Qiagen RNeasy Mini Kit, including on-column DNase digestion, and yield and purity were assessed using a NanoDrop 1000 spectrophotometer (Thermo Scientific, Waltham, MA, USA). Ribosomal RNA was depleted with the NEBNext rRNA Depletion Kit (New England Biolabs, Ipswich, MA, USA), and RNA fragmentation, double-stranded cDNA synthesis, and adaptor ligation were performed using the NEBNext Ultra II Directional RNA Library Prep Kit (New England Biolabs, Ipswich, MA, USA). Libraries were PCR-enriched, quantified with PicoGreen (Thermo Fisher Scientific, Waltham, MA, USA), pooled equimolarly, quality-checked on the Agilent Tapestation 2200, and quantified by qPCR. Libraries were diluted to 200 pM with 1% PhiX spike-in and sequenced on an Illumina NovaSeq 6000 (2 × 50 bp paired-end reads), targeting at least 20 million reads per sample (TTU Center for Biotechnology and Genomics).

For RNA-seq data processing, BCL files from mouse liver tumors were demultiplexed and converted to FASTQ format using the Illumina bcl2fastq utility in BaseSpace Sequence Hub. FASTQ quality was assessed with FastQC (https://www.bioinformatics.babraham.ac.uk/projects/fastqc/, accessed on 23 January 2026), and paired-end reads were subsequently trimmed and corrected using Trimmomatic with default parameters [41]. RNA-seq transcriptomes were aligned to the mouse reference genome GRCm39 using the STAR aligner [42] and raw read counts were obtained using featureCounts [43]. TPM values were calculated by first dividing read counts by gene length in kilobases to obtain reads per kilobase (RPK), then summing all RPKs in a sample and dividing by 1 million to obtain a scaling factor, and finally dividing each RPK by this scaling factor to yield TPM.

### 2.6. Sequence Homology and Identity Between Human and Mouse MAGE Genes

Databases of human and mouse *MAGE* genes were constructed by querying the NCBI Gene database (August 2025) using the search terms MAGE[All Fields] AND “Mus musculus”[Organism] AND alive[prop] and MAGE[All Fields] AND “Homo sapiens”[Organism] AND alive[prop]. Results were compared with our previously curated *MAGE* gene database [26], excluding non-*MAGE* genes and pseudogenes. Orthology relationships and protein sequence identities were obtained using Ensembl BioMart (release 114). Mouse *Mage* genes were queried by NCBI Gene ID to identify human orthologs, recording the reported percentage identity; when multiple orthologs had comparable identities, the mean was used. The procedure was repeated reciprocally using human *MAGE* genes to identify mouse orthologs. Percentage identities of *MAGE* homologs are listed in Table S2.

### 2.7. Analysis of the Cancer Genome Atlas (TCGA) Data

A comprehensive analysis was performed on the Liver Hepatocellular Carcinoma (TCGA-LIHC) dataset. mRNA expression data, along with clinical and sample information, were retrieved from the Cancer Data Gateway (CDG) portal (https://portal.gdc.cancer.gov/projects/TCGA-LIHC) on 8 August 2024. In addition to analyzing *MAGE* gene expression in tumor and healthy samples, the relationship between *MAGE* gene expression and clinical variables, such as age and sex, was explored.

To classify samples into high and low expression groups, an exponential distribution was fitted to the TPM values of each *MAGE* gene that exceeded a minimum expression threshold. Based on the fitted distribution, 10,000 random samples were generated and clustered using the DBSCAN algorithm, with an epsilon (eps) of 0.1 and a minimum sample size of 5. The two largest resulting clusters were compared, and the lowest of the two was selected as the optimal cutoff. Samples with a *MAGE* TPM value greater than or equal to the cutoff were assigned to the high expression group; otherwise, they were classified as low expressers. The code implementing this method is available at https://github.com/jidiaz/TCGA-TTU/blob/main/TCGA2-Jorge/240322_TCGA_TC3R_Overview-Jorge.ipynb, accessed on 23 January 2026.

Using these optimal cutoff values to divide the dataset into high- and low-expression groups for each MAGE gene, survival curves were estimated using the Kaplan-Meier method, and differences between expression groups were initially evaluated using the log-rank test. Censored observations (patients alive at last follow-up) were appropriately handled using right-censoring. Then, multivariate Cox regression analysis was performed to adjust for age, sex, and tumor stage. Gene expression values (TPM) were log2-transformed (log2[TPM + 1]) and standardized (z-score normalization) prior to analysis. Clinical covariates were processed as follows: age was standardized to improve model convergence; sex was encoded as a binary variable (male = 1, female = 0); and tumor stage was encoded ordinally (Stage I = 1, Stage II = 2, Stage III = 3, Stage IIIA = 4, Stage IIIB = 5, Stage IIIC = 6, Stage IV = 7, Stage IVA = 8, Stage IVB = 9). For each gene, we fitted two Cox models: (1) a univariate model with gene expression as the sole predictor to obtain unadjusted hazard ratios (HRs) and (2) a multivariate model including gene expression, age, sex, and stage to obtain adjusted HRs. We report both unadjusted and adjusted HRs with 95% confidence intervals (CIs), corresponding *p*-values, and concordance indices (C-index) to assess model discrimination. The proportional hazards assumption was tested using Schoenfeld residuals. Multiple testing correction was applied using the Benjamini-Hochberg false discovery rate (FDR) method. All analyses were performed using the lifelines package (v0.27+) in Python (v3.12.11), with a minimum requirement of 30 samples and 10 events per gene for stable parameter estimates.

### 2.8. Differential Expression Analysis (DEA)

First, using the method described in the TCGA analysis section, we stratified the two datasets (i.e., from TCGA and from our RNA-seq of mouse liver tumors) into low- or high-*MAGED2*-expressing groups with a cutoff of 32.24 TPM for TCGA data or 5.25 TPM for mouse RNA-seq data. Differential expression analysis was then performed with the raw counts using the DESeq2 (https://genomebiology.biomedcentral.com/articles/10.1186/s13059-014-0550-8, accessed on 23 January 2026) package, identifying differentially expressed genes (DEGs) based on an adjusted *p*-value threshold of 0.05 and an absolute fold change of ≥1. Then, over-representation analysis was performed with the human or mouse DEGs, divided into up- or down-regulated groups, using the clusterProfiler package in RStudio (version 2025.05.0+496 “Mariposa Orchid”).

To test whether the overlap of DEGs between human and mouse exceeded what would be expected by chance, we used Fisher’s Exact Test. All genes detected in both species were used as the background universe (N = 25,363). Among these, 31 DEGs were shared between human DEGs (n = 283) and mouse DEGs (n = 2204).

### 2.9. Plotting and Statistical Analyses

**Figure 2:**
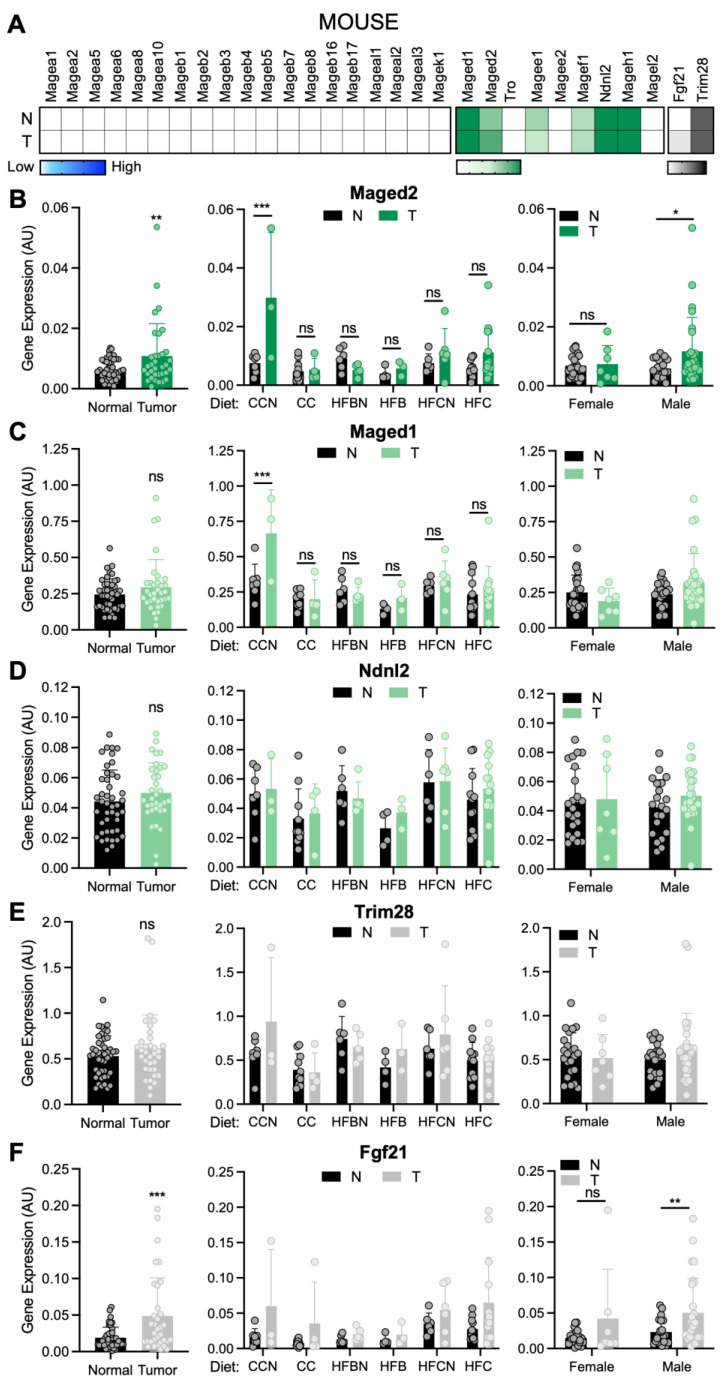
Expression of *Mage* genes in mouse liver tumors. (**A**) Heatmap shows the average values for all normal liver and liver tumor samples from mice (n = 78). (**B**–**F**) Graphs show the expression levels (mean ± SD with circles indicating individual samples) of (**B**) *Maged2*, (**C**) *Maged1*, (**D**) *Ndnl2*, (**E**) *Trim28*, and (**F**) *Fgf21* as determined by RT-qPCR for (left graphs) all normal liver (n = 44) and liver tumor samples (n = 34). Expression is shown for indicated genes in normal liver and liver tumors divided by (middle graphs) diet (CCN: n_normal_ = 7, n_tumor_ = 3; CC: n_normal_ = 10, n_tumor_ = 4, HFBN: n_normal_ = 6, n_tumor_ = 5; HFB: n_normal_ = 4, n_tumor_ = 3; HFCN: n_normal_ = 6, n_tumor_ = 6; HFC: n_normal_ = 11, n_tumor_ = 13) and (right graphs) gender (Female: n_normal_ = 23, n_tumor_ = 7; Male: n_normal_ = 21, n_tumor_ = 27). Statistical significance was determined by (left graphs) unpaired *t* test to compare expression between normal and tumor or by (middle and right graphs) two-way ANOVA followed by Šídák’s multiple comparisons test to compare expression between diet groups or gender for each gene [*p* ≤ 0.05 (∗), *p* ≤ 0.01 (∗∗), *p* ≤ 0.001 (∗∗∗), *p* ≥ 0.05 (non-significant, ns, or no asterisks)]. Control chow + AHE (CCN), control chow (CC), high-fat beef + AHE (HFBN), high-fat beef (HFB), high-fat casein + AHE (HFCN), high-fat casein (HFC), ammonium hydroxide enhancement (AHE).

**Figure 3:**
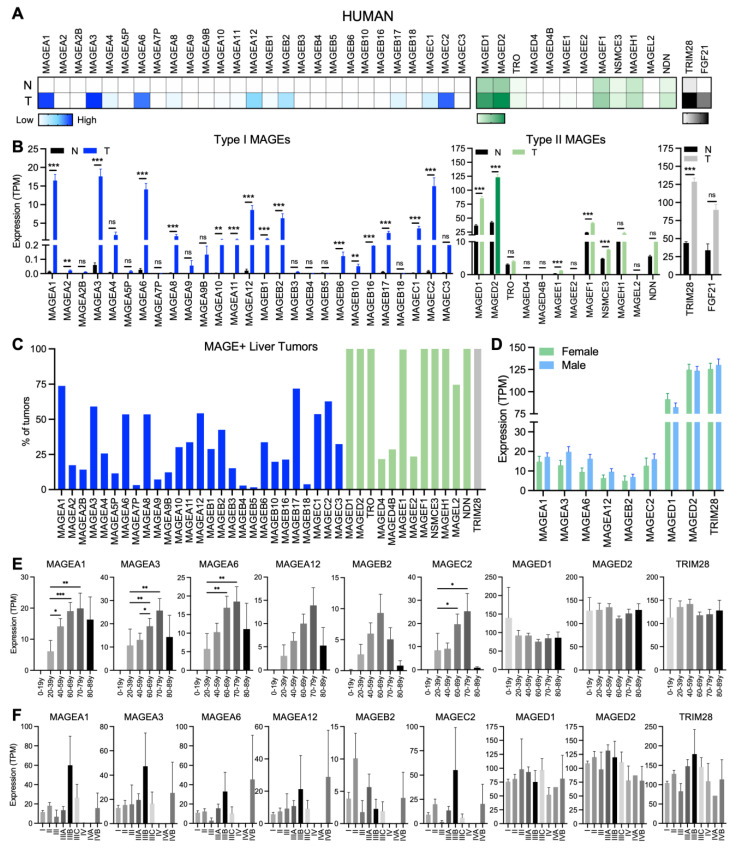
Expression of *MAGE* genes in human liver tumors. (**A**) Heatmap shows the average TPM values for all normal liver and HCC samples from humans. (**B**) Graphs show the expression levels (mean ± SEM) of (left) type I *MAGEs*, (middle) type II *MAGEs*, and (right) *TRIM28* and *FGF21* in normal liver (n = 50) and HCC samples (n = 374). (**C**) Graph shows the percentage of HCC tumors that expressed the indicated *MAGEs*. The expression of select *MAGEs* and *TRIM28* in HCC is graphed according to (**D**) sex (n_female_ = 121, n_male_ = 253), (**E**) age (n_0–19y_ = 3, n_20–39y_ = 29, n_40–59y_ = 137, n_60–69y_ = 122, n_70–79y_ = 65, n_80–89y_ = 14), and (**F**) pathological stage (n_stage I_ = 191, n_stage II_ = 98, n_stage III_ = 6, n_stage IIIA_ = 73, n_stage IIIB_ = 8, n_stage IIIC_ = 10, n_stage IV_ = 3, n_stage IVA_ = 1, n_stage IVB_ = 2). Statistical significance was determined by (**B**,**D**) multiple Mann-Whitney tests with two-stage step-up (Benjamini, Krieger, and Yekutieli) multiple comparisons FDR method to compare gene expression between either (**B**) normal tissues and tumors or (**D**) male and female HCC samples or by (**E**,**F**) Kruskal-Wallis tests followed by Dunn’s multiple comparisons test to compare expression among (**E**) age groups or (**F**) tumor stages for each gene [*p* ≤ 0.05 (∗), *p* ≤ 0.01 (∗∗), *p* ≤ 0.001 (∗∗∗), *p* ≥ 0.05 (non-significant, ns, or no asterisks)]. All data are from TCGA-LIHC, downloaded on 8 August 2024.

Heatmaps and plots were made in GraphPad Prism 10 (version 10.4.2). Kaplan-Meier plots were generated in Python (v3.12.11) using the Matplotlib (v3.10.0) library. Diagrams were made in BioRender (www.BioRender.com, last accessed on 15 January 2026). Results in Figure 2 are expressed as the mean ± standard deviation (SD), with individual data points indicated, and results in Figure 3 are expressed as the mean ± standard error of the mean (SEM). To determine the appropriate statistical methods, the data was first tested for normality in GraphPad Prism 10 using the D’Agostino-Pearson omnibus normality test and quantile-quantile (QQ) plots. Since the RT-qPCR data were continuous and approximately normally distributed, statistical significance was determined by parametric tests, either Student’s *t*-test, one-way ANOVA, or two-way ANOVA, followed by Dunnett’s multiple comparisons test for all samples compared to the control. Since the RNA-seq TCGA data was continuous but not normally distributed, statistical significance was determined by nonparametric tests: multiple Mann-Whitney tests with the two-stage step-up (Benjamini, Krieger, and Yekutieli) multiple comparisons FDR method. For all analyses, *p*-values ≤ 0.05 were considered statistically significant [*p* ≤ 0.05 (∗), *p* ≤ 0.01 (∗∗), *p* ≤ 0.001 (∗∗∗), *p* ≥ 0.05 (non-significant, ns, or no asterisks)].

## 3. Results

### 3.1. Mage Gene Expression in Mouse Liver Tumors

**Figure 1:**
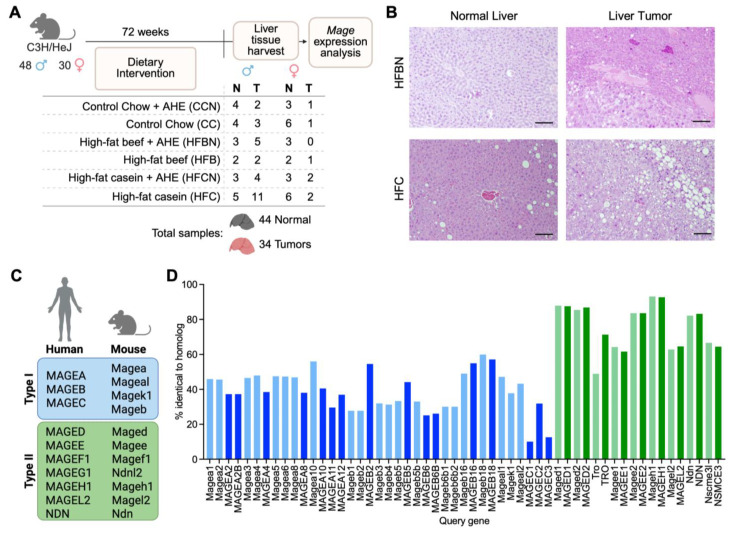
Comparison of human and mouse *MAGE* genes. (**A**) Experimental scheme. (**B**) Normal liver and liver tumor samples from mice fed either an HFBN or HFC diet were stained with H&E and imaged. Scale bar = 200 µm. (**C**) The *MAGE* gene family in humans and mice, divided into type I and type II subfamilies. (**D**) Graph showing the percentage of sequence identity between human and mouse *MAGE* gene homologs. Mouse query genes are shown in light blue and green, with human query genes in dark blue and green. Orthology relationships and sequence identity values were obtained from Ensembl BioMart (release 114).

In this study, we examined *Mage* gene expression in normal and tumor liver tissues from 78 C3H/HeJ mice subjected to a high-fat diet combined with dietary protein supplementation and AHE, modeling chronic diet-induced obesity, a known cancer risk factor (Figure 1A). C3H/HeJ mice were chosen because both males and females in this strain are susceptible to obesity [36,37]. High-fat diets increased liver tumor incidence in male mice, whereas diet had little impact on tumor development in females (Table 1). Survivability curves and complete liver tumor counts for all diets, except the CCN group, were previously published [36,37]. Male mice on the HFC diet showed the highest tumor incidence (11/78; 14%) (Figure 1A, Table 1). Histopathological evaluation of liver sections from an HFC-fed male revealed moderate mid-zonal to periportal micro- and macrovesicular steatosis in the normal liver, consistent with a high-fat diet, and neoplastic cells with morphology characteristic of HCC in tumors (Figure 1B, Table S1).

Humans and mice have ∼40 *MAGE* genes that are categorized as type I or type II based on their sequence homology, tissue expression pattern, and chromosomal location [20,26]. Type I *MAGE* genes include members of the *MAGE-A* and *-B* families, the primate-specific *MAGE-C* family, and the mouse-specific *Mage-a–like* (*Mage-al* and *Mage-k1*) family (Figure 1C). Type II *MAGEs*, consisting of the *MAGE-D*, -*E*, -*F*, -*G*, -*H*, -*L*, and *NECDIN* genes, are more ubiquitously expressed in humans and mice and not typically associated with cancer. Between humans and mice, the type II *MAGE* genes have higher percentages of sequence identity than the type I *MAGEs* (Figure 1D and Table S2). *MAGE* genes in healthy humans and mice show similar expression patterns: type I *MAGE* genes are restricted to or highly enriched in the testis, whereas type II *MAGE* genes are broadly expressed, with enrichment in the brain [26]. In humans, the type I *MAGEs* are called CTAs because of their normal expression in the testes and aberrant activation in several cancers; however, it is not clear whether these *Mage* genes are also expressed in mouse tumors.

To address this question, we analyzed the mRNA expression levels of *Mage* genes in normal liver tissue and in liver tumors from mice maintained on different diets (Figure 1A and Figure S1). In our early RNA-seq analysis from a partial set of samples (n = 60), we observed little to no expression of type I *Mages* in normal livers and tumors (Figure S2). Interestingly, liver tumors exhibited a slight increase in the more ubiquitously expressed type II *Maged2*, though not statistically significant (Figure S2B). With the full set of mouse liver samples (n = 78), we measured the expression of all mouse *Mage* genes by RT-qPCR, using our previously validated and specific primers [26]. Consistent with the RNA-seq results, RT-qPCR analysis confirmed that type I *Mage* expression was below the detection limit in mouse liver tumors under the conditions tested (Figure 2A). This finding may reflect species-specific regulation and a limited role for these genes in murine HCC. In contrast, *Maged2* mRNA was significantly upregulated in mouse liver tumors (*p* = 0.02, Cohen’s d = 0.5, 95% CI [0.0008–0.0120]) (Figure 2B), coinciding with the recent reports of *MAGED2* as a negative prognostic factor in human HCC [12,44]. Among the diet groups, *Maged2* expression was significantly increased in mouse liver tumors from the CCN diet group (*p* < 0.0001, Cohen’s d = 1.9, 95% CI [0.1043–0.5795]), although no significant differences in body weight were observed between animals with and without tumors (Figure S2E). We also observed a statistically significant increase in *Maged1* expression in liver tumors from mice fed a CCN diet (*p* = 0.0014, Cohen’s d = 1.8, 95% CI [0.0344–0.0102]) (Figure 2C). This group of mice also exhibited the lowest body weight among animals that developed tumors, reaching statistical significance compared with the HFBN group (Figure S2E). Other type II *Mages*, like *Ndnl2*, were expressed at similar levels, regardless of normal liver or liver tumor, diet, or sex (Figure 2D). We also measured *Trim28*, as higher *TRIM28* expression levels have been found in HCC and other cancers, and TRIM28 is a molecular partner of MAGEA2, -A3/6, and -C2 proteins in human cancers, including in HCC [33,45,46,47]. However, *Trim28* expression did not differ between normal liver and tumor samples in mice (Figure 2E). In addition, we measured fibroblast growth factor 21 (*Fgf21*) expression levels since FGF21 is a hepatokine and a metabolic regulator that has been suggested as a biomarker for liver tumors [48,49,50,51]. The mouse liver tumors had elevated *Fgf21* expression levels compared to normal livers (*p* = 0.0006, Cohen’s d = 0.8, 95% CI [0.0133–0.0461) (Figure 2F) [51,52].

In mice with hepatocyte-specific knockout (LKO) of the cholesterogenic enzyme lanosterol 14α-demethylase (*Cyp51*), which causes chronic disruption of late cholesterol synthesis and lanosterol accumulation, liver tumors develop with a female-to-male prevalence ratio of approximately 2:1 [38,39]. The *Cyp51* LKO mice on a normal diet remained lean with an increased liver/body weight ratio, especially in 24-month-old female *Cyp51* LKO mice, which had the highest incidence of liver tumors [38,39]. RT-qPCR analysis of spontaneous liver tumors from *Cyp51* LKO mice (n = 11) further confirmed that type I *Mage* genes were below the limit of detection, indicating that they are not activated in mouse tumors (Figure S3). Expression levels of *Maged1* and *Trim28* were higher in liver tumors from *Cyp51* LKO mice compared to liver tissue from WT mice, although the differences did not reach statistical significance (Figure S3B). We also analyzed the *Maged1* and *Maged2* hepatic expression from two microarray datasets (GSE58271 and GSE127772) but did not note any changes in their expression levels between diet groups, nor normal vs. tumor in WT and *Cyp51* LKO mice [39,53]. These data suggest that the overexpression of *Maged1* and *Maged2* may not be connected to the cholesterol-related HCC pathogenesis in mice.

### 3.2. Expression of MAGEs in Human HCC

To compare our findings from mouse liver tumors to human HCC, we analyzed *MAGE* expression data from the TCGA Liver Hepatocellular Carcinoma dataset. Consistent with previous studies, the type I *MAGEs* are expressed in HCC but not normal liver (Figure 3A,B) [2,30,33,54,55]. Many type II *MAGE* genes are expressed in all human HCCs; however, the expression levels of *MAGED1* and *MAGED2* were higher than those in normal liver in 75% and 95% of HCC samples, respectively (Figure 3A–C). Expression of the selected type I and II *MAGE* genes in HCC samples was not significantly different between male and female patients (Figure 3D). Similarly, the expression of *MAGEA12*, *MAGEB2*, *MAGED1*, and *MAGED2* in HCC samples was not significantly different among HCC samples from patients of different age groups (Figure 3E). In contrast, *MAGEA1*, -*A3*, -*A6*, and -*C2* expression did show some statistically significant differences among the age groups (Figure 3E) and stage of HCC samples (Figure 3F). Although type I *MAGEs* tended to show the highest expression at stage IIIb, the expression of type II *MAGED1* and *MAGED2* genes was not significantly different among HCC stages (Figure 3F).

**Figure 4:**
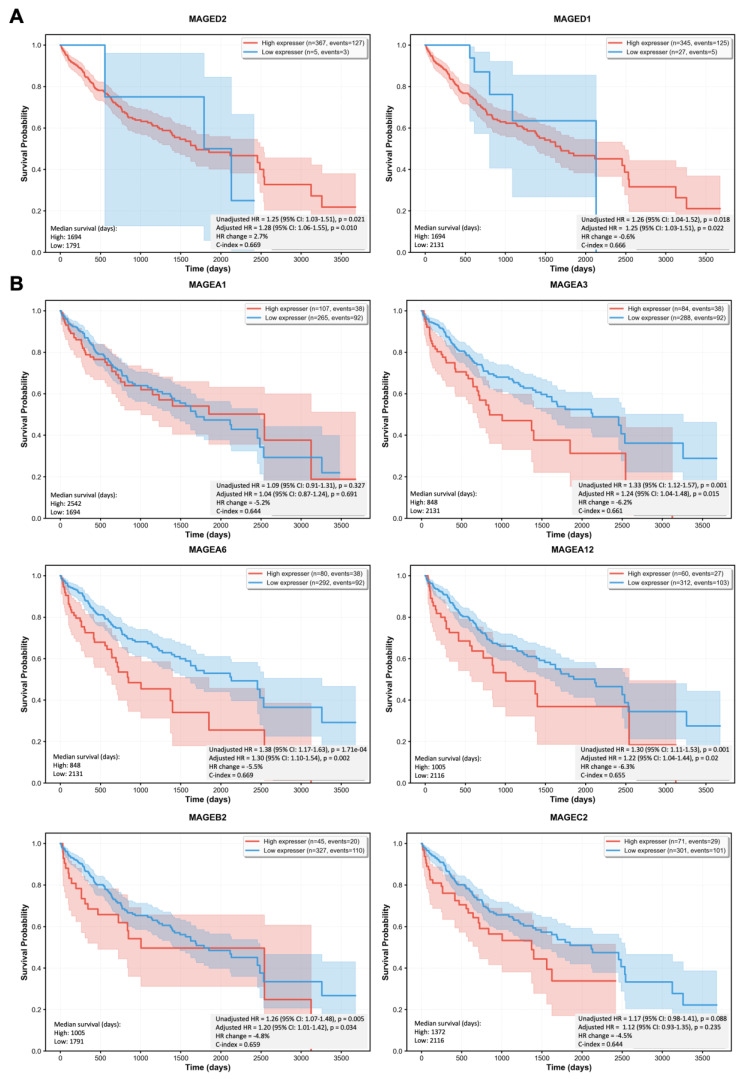
Kaplan-Meier plots show the probability of survival based on low (blue line) or high (red line) expression of (**A**) type II *MAGEs* or (**B**) type I *MAGEs*. After determining optimal cutoff values (see Section 2.7) to divide the dataset into high- and low-expression groups for each *MAGE* gene, survival curves were estimated using the Kaplan-Meier method, and differences between expression groups were initially evaluated using the log-rank test. Censored observations (patients alive at last follow-up) were appropriately handled using right-censoring. Multivariate Cox regression analysis was performed to adjust for age, sex, and tumor stage. For each gene, we fitted two Cox models: (1) a univariate model with gene expression as the sole predictor to obtain un-adjusted hazard ratios (HRs) and (2) a multivariate model including gene expression, age, sex, and stage to obtain adjusted HRs. We report both unadjusted and adjusted HRs with 95% confidence intervals (the shaded red and blue areas are based on the unadjusted HR), corresponding *p*-values, and concordance indices (C-index) to assess model discrimination. The proportional hazards assumption was tested using Schoenfeld residuals. Multiple testing correction was applied using the Benjamini-Hochberg FDR method. All analyses were performed using the lifelines package (v0.27+) in Python (v3.12.11), with a minimum requirement of 30 samples and 10 events per gene for stable parameter estimates.

To determine the prognostic value of *MAGED1* and *MAGED2* genes in HCC, we performed Kaplan–Meier survival analyses followed by multivariate Cox proportional hazards regression. Patients with high *MAGED2* expression had a 28% higher risk of death compared with those with low expression [adjusted hazard ratio (HR) = 1.28, 95% CI: 1.06–1.55, *p* = 0.010] (Figure 4A). Notably, the effect size increased after adjustment (ΔHR = +2.7%), indicating that *MAGED2* provides prognostic information independent of traditional clinical factors. Similarly, patients with high *MAGED1* expression had a 25% increased risk of death [adjusted HR = 1.25 (95% CI: 1.03–1.51), *p* = 0.022]. Consistent with previous studies, our analyses also show that high expression of *MAGEA3*, *MAGEA6*, or *MAGEA12* in HCC is associated with reduced survival probability (Figure 4B) [12]. Patients with high *MAGEB2* expression exhibited a 20% increased risk of death, although this effect was weaker relative to other *MAGEs* and only marginally significant [adjusted HR = 1.20 (95% CI: 1.01–1.42), *p* = 0.034]. In contrast, *MAGEA1* and *MAGEC2* did not show prognostic applicability in our dataset, despite previous reports describing associations with HCC clinical stage and differentiation [12].

**Figure 5:**
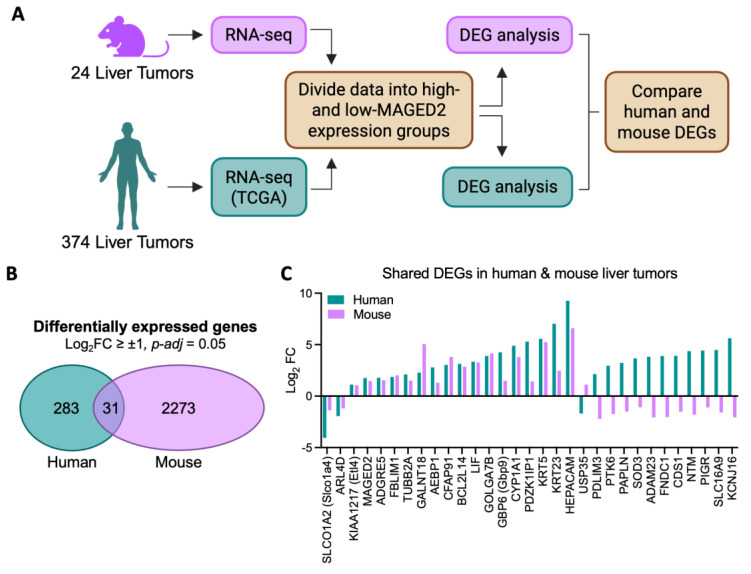
Differential expression analysis (DEA) of mouse liver tumors and human HCC. (**A**) Schematic shows how the analysis was conducted to determine DEGs in human and mouse liver tumors with high *MAGED2* expression. (**B**) Venn diagram illustrates that some DEGs are common between human and mouse liver tumors with high *MAGED2* expression. (**C**) The graph shows upregulated and downregulated DEGs shared between human and mouse liver tumors with high *MAGED2* expression (Fisher’s exact test, OR = 1.29, *p* = 0.1074).

Finally, we analyzed RNA-seq data to identify differentially expressed genes (DEGs) between liver tumors with low or high *MAGED2* expression in both humans and mice (Figure 5A). Among the 2273 DEGs from mouse tumors and 283 DEGs from human tumors (Table S3) (Log2FC ≥ ±1 and *p*-adj = 0.05), 31 genes overlapped between the two species (Figure 5B). Of these 31 DEGs, 17 genes were upregulated and two were downregulated in both humans and mice, while the remaining 12 genes showed opposite expression changes in humans and mice (Figure 5C). The overlap of 31 genes showed a slight enrichment, with an odds ratio of 1.29, but this did not reach statistical significance (*p* = 0.1074), suggesting that the observed overlap could occur by chance. However, similarities between human and mouse liver transcriptomes are generally limited, with interspecies differences larger than those observed between disease stages in fatty liver disease models, for example [8]. Therefore, a shared DEG list between *Maged2*-positive tumors may have biological relevance and warrants further investigation.

Some of the shared upregulated DEGs between the two species in *MAGED2*-high tumors are genes that are overexpressed in HCC or other cancers (*AEBP1* [56], *CYP1A1* [57], *FBLIM1* [58], *KRT5*, *BCL2L14*, *TUBB2A*, and *GALNT18*), as well as some genes associated with worse prognosis in HCC (*ADGRE5* and *KRT23*) [59,60,61,62,63,64,65,66,67]. *SLCO1A2*, a member of the organic anion-transporting polypeptide (OATP) family involved in the uptake and distribution of endogenous molecules and xenobiotics, including bile acids, was downregulated in *MAGED2*-high tumors in both humans and mice. Although the role of *SLCO1A2* in HCC is less well defined, other SLCO family members are consistently downregulated in HCC and have been proposed as prognostic markers. The reduced expression of *SLCO1A2* in *MAGED2*-high tumors across species therefore suggests that elevated *MAGED2* may be associated with a more aggressive tumor phenotype in liver tumors of both species [68]. Further studies are needed to better understand the mechanism of action of *MAGED2* and to determine whether *MAGED2* is regulated by high-fat or other types of diets.

We also analyzed all human and mouse DEGs separately and found several enriched GO terms across all three ontologies (Figures S4 and S5). Interestingly, while the downregulated biological pathways in human *MAGED2*-high tumors were predominantly associated with oxidative metabolism (Figure S5), those in mice were enriched for xenobiotic metabolism (Figure S4). This result likely reflects known species-specific differences in liver transcriptomes and drug-metabolism gene expression [69,70]. The major upregulated pathways also differed between species: cell-cycle and proliferative programs were most prominent in mouse tumors, whereas extracellular matrix–related pathways were most enriched in human tumors.

## 4. Discussion

Though infection by hepatitis B or C virus is the main risk factor for HCC development, the prevalence of modifiable risk factors (e.g., alcohol consumption and metabolic syndrome) is on the rise [4,71]. Obesity is a major contributor to the development of metabolic syndrome, MASLD/MASH, and HCC [36,72]. Advanced HCC has limited treatment options, and early diagnostic markers and novel therapies are needed. Emerging immunotherapies, alone or in combination, are transforming systemic HCC treatment; however, prognostic markers and targets to guide therapy and predict outcomes need to be identified [73]. Tumor antigens, including *MAGEs*, are promising targets of solid tumor immunotherapy [74], but little is known about their expression and role in mouse tumors. Although there is no perfect preclinical model to fully recapitulate human disease, several mouse models of HCC were essential for uncovering disease pathogenesis, identifying molecular targets, and developing mechanistically based therapeutic interventions [75]. Herein, we studied *Mage* expression in spontaneous liver tumors from mice fed different high-fat diets in a chronic diet-induced obesity mouse model and in a genetic model of dysregulated cholesterol biosynthesis.

A recent study found that dietary protein source and its pH, modified by AHE, are influential factors in the longevity of C3H/HeJ mice, dependent on sex [36]. Female mice fed diets with AHE (i.e., HFCN and HFBN) showed improved longevity regardless of the protein source (i.e., beef or casein), while increased longevity was only observed in male mice fed HFBN diets [36,76]. Among all dietary groups, the tumor incidence between the sexes was almost even [36]. However, tumors in male mice developed almost exclusively in the liver, with the highest incidence in the HFC dietary group, which is in line with the higher incidence of HCC in men [36,77]. Male mice fed an HFC diet also had the highest incidence of liver tumors in our study (Figure 1A and Table 1). In contrast, tumors in female mice more frequently developed in the ovaries, mammary tissue, or lungs, with liver tumors having the lowest incidence [36].

Chronic cholesterol synthesis block in *Cyp51* LKO mice was previously shown to lead to liver tumor development [39]. While CYP51 normally converts lanosterol into cholesterol precursors by removing a methyl group at the 14α position, tissue-specific regulation of cholesterol biosynthesis can cause sterol intermediate accumulation in tissues, such as gonads and brain. In the liver, however, cholesterol synthesis is tightly controlled [78,79,80]. Chronic blockade of this pathway in *Cyp51* LKO mice induces female-predominant hepatocarcinogenesis during aging, paralleling the increased risk of HCC in women after menopause and highlighting this model’s relevance for human disease [39].

Previous studies of *MAGE* gene expression in human HCC have established that several type I *MAGEs* are aberrantly expressed in these liver tumors. Particularly, high expression of *MAGEA9* in HCC indicates unfavorable survival and contributes to cancer malignancy and chemoresistance [18,31]. *MAGEA1* and *MAGEC2* are highly correlated with HCC clinical stage and differentiation, and *MAGEA6*, -*A10*, -*A12*, -*B4*, and -*C3* are correlated with HCC prognosis [12]. MAGE–TRIM28-mediated degradation of FBP1 was shown to drive reprogramming of cancer cell metabolism and HCC tumorigenesis [25,33]. Contrary to the published evidence showing aberrant expression of type I *MAGE* genes in human liver tumors [2,18,29,31,81,82], in our study, these *Mage* genes were not expressed above the limit of detection in mouse liver tumors, regardless of dietary group. These results suggest that neither diet nor oncogenic transformation activates aberrant expression of type I *Mage* genes in mouse liver tumors. This finding is consistent with the possibility of species- and tissue-specific oncogenic activation of type I *MAGE* genes, as we have also observed in pediatric medulloblastomas and corresponding genetic mouse models [21,83]. Indeed, other CTAs, such as *PAGE4* and *FATE1*, are expressed in a species- and tissue-specific manner [84,85]. Promoter hypermethylation has been shown to underlie the restricted expression of tumor-associated *MAGE* antigens in human adult tissues [86,87], while tumor-associated hypomethylation drives their activation in solid cancers [88,89]. However, the transcriptional details that determine the cancer-type-specific expression of individual *MAGE* family members, and how promoter regulation differs in mouse tumors, remain almost completely unknown. Interestingly, it was recently shown that another cancer-testis antigen, *BORIS* (*CTCFL*), a testis-specific paralog of the chromatin organizer CTCF, also shows limited conservation between humans and mice, despite being present in both species. Unlike the strictly testis-specific expression of mouse *Boris*, human *BORIS* escapes complete somatic silencing, resulting in mosaic expression in a subset of mouse somatic cells. This ectopic expression is associated with reduced survival, increased tumor incidence, and a shift in tumor spectrum toward aggressive lymphomas [90]. Similarly, expression of human *MAGEA4* in mice has been reported to induce lung tumors in mouse models [91]. Future investigation into promoter methylation, chromatin accessibility, and transcription-factor binding is necessary and would provide insight into the underlying regulatory differences in human and mouse *MAGE* expression.

Diet may affect the expression of type II *Mages*, as *Maged1 and Maged2* expression levels were significantly higher in tumors from mice in the CCN diet group and trended upward in the HFC and HFCN groups, while the levels were similar between normal liver and tumors in mice from the CC and HFB groups. In addition to diet-dependent differences in *Maged1* and *Maged2* mRNA expression in liver tumors, we found that these genes were not activated in tumors from *Cyp51* LKO mice, which accumulate pre-cholesterol sterols due to impaired cholesterol biosynthesis [38,39]. These findings suggest that *Maged1* and *Maged2* mRNA overexpression may not be associated with lipid-driven HCC pathogenesis in mice but may instead be linked to the enhancement of dietary protein sources with AHE or casein. Notably, AHE has recently been proposed as a potentially health-beneficial food-processing modification that impacts liver metabolism in a sex-specific manner [92]. For example, diets containing AHE decreased expression of CYP3A4 protein, a major hepatic cytochrome P450 enzyme involved in drug metabolism and xenobiotic detoxification, in males, coinciding with a statistically significant increase in *Maged2* mRNA expression (Figure 2B). MAGED2 has previously been implicated in Bartter syndrome [93] and, at the molecular level, has been shown to act as a master regulator of cellular cAMP signaling under conditions of cellular stress, including hypoxia [94]. Interestingly, *Maged1*-deficient mice develop progressive obesity [95]. Additional research is needed to understand how *MAGED1* and *MAGED2* are connected to sex, diet,—particularly fat and protein content and quality—obesity, and liver cancer.

Though most earlier studies only looked at the type I *MAGEs* in HCC, a few recent studies have also found increased expression of type II *MAGEs* in liver tumors. One study found overexpression of *MAGED4* mRNA in HCC cell lines and surgically removed tumor samples from Japanese patients, suggesting that *MAGED4* may serve as a predictive biomarker of early recurrence and mortality [96]. However, *MAGED4* is human-specific and is not present in the mouse genome [25,26]. In a subsequent study, the expression of *MAGED2* was higher in 44% of HCC tissues from patients, and these patients had significantly shorter survival time, indicating that *MAGED2* may serve as a prognostic indicator following curative resection [44]. A comprehensive analysis of *MAGE* expression in HCC from publicly available databases revealed that several *MAGE* genes, including *MAGED1* and *MAGED2*, are dysregulated in HCC and correlate with clinicopathological parameters (i.e., clinical stage, differentiation, and prognosis) [12]. It was previously shown that *MAGED2* is involved in cell adhesion and metastasis of tumor cells in colorectal cancer and acts as a regulator of breast cancer cell motility [97,98]. However, whether *MAGED2* is a driver or a consequence of tumorigenesis in HCC has not been elucidated. Together with our data, these results suggest that *MAGED2* may contribute to liver cancer in both humans and mice, supporting the promise of using the mouse model for further mechanistic studies of *MAGED2*’s role in HCC. Although *MAGED2* upregulation has been reported in HCC patients, its dysregulation in mice, particularly in the context of diet-induced HCC, has not been previously examined. These first insights open the door for more detailed mechanistic studies. Future functional analyses employing both loss- and gain-of-function approaches will be essential to define the molecular role of MAGED2 in HCC progression. Given that the differences between human and mouse transcriptomes were found to be larger than the differences between disease stages of MASLD models, with MASLD being a hepatic manifestation of obesity and a major risk factor for liver cancer, a shared marker such as *Maged2* may be important [99]. Our findings emphasize the importance of comparative, cross-species (“One Health”) approaches in cancer research for selecting appropriate models and accurately interpreting results.

We acknowledge that our results are based on gene expression data; therefore, Western blot analysis or immunohistochemistry (IHC) would be valuable to confirm protein levels. Notably, studies in human liver and breast cancer have demonstrated a correlation between increased mRNA and corresponding protein levels [44,100]. Furthermore, functional analyses to elucidate the mechanisms underlying diet-induced *MAGED2* activation or its function were not performed in the present study. The findings reported here may inform future studies aimed at evaluating the prognostic and therapeutic potential of MAGED2, as well as other MAGE proteins; however, further studies are warranted to determine the species specificity of type I *MAGE* activation and the potential shared functions of type II *MAGED1* and *MAGED2* in normal liver physiology and liver pathogenesis.

## 5. Conclusions

In this study, we (1) evaluated diet-induced expression of type I and II *Mage* genes in normal livers and liver tumors from male and female C3H/HeJ mice for the first time, (2) discovered that type I *Mage* genes are not expressed above the limit of detection in mouse liver tumors, and (3) demonstrated upregulation of *Maged2* in mouse liver tumors. These data are helpful for further investigation into the mechanistic roles of MAGE proteins in cancer. Our findings suggest that endogenous mouse tumors may not always be suitable models, particularly when targeting cancer-testis antigens in cancer immunotherapy, underscoring the importance of characterizing *Mage* expression patterns. In contrast, the common upregulation of *MAGED2* suggests that it is a conserved marker of liver tumors in both humans and mice and warrants further investigation of its role in HCC pathogenesis using mouse models.

## Figures and Tables

**Table 1:** Percentage of mice with normal livers or liver tumors in each diet group, stratified by sex.

	Normal Liver		Liver Tumor		
Group ^1^	Female	Male	Female	Male	Total
CCN	3 (4%)	4 (5%)	1 (1%)	2 (3%)	10 (13%)
CC	6 (8%)	4 (5%)	1 (1%)	3 (4%)	14 (18%)
HFBN	3 (4%)	3 (4%)	0 (0%)	5 (6%)	11 (14%)
HFB	2 (3%)	2 (3%)	1 (1%)	2 (3%)	7 (9%)
HFCN	3 (4%)	3 (4%)	2 (3%)	4 (5%)	12 (15%)
HFC	6 (8%)	5 (6%)	2 (3%)	11 (14%)	24 (31%)
Total	23 (29%)	21 (27%)	7 (9%)	27 (35%)	78 (100%)

^1^ Control chow + AHE (CCN), control chow (CC), high-fat beef + AHE (HFBN), high-fat beef (HFB), high-fat casein + AHE (HFCN), high-fat casein (HFC), ammonium hydroxide enhancement (AHE).

## Data Availability

The raw data supporting the conclusions of this article will be made available by the corresponding authors upon reasonable request. The TCGA-LIHC data was retrieved from the Cancer Data Gateway (CDG) portal [https://portal.gdc.cancer.gov/projects/TCGA-LIHC, accessed on 23 January 2026]. The microarray datasets (GSE127772 and GSE58271) for Figure S2 were retrieved from GEO [https://www.ncbi.nlm.nih.gov/geo/] on 18 March 2025.

## Supplementary Materials

The following supporting information can be downloaded at https://www.mdpi.com/article/10.3390/cimb48020139/s1.

## Abbreviations

The following abbreviations are used in this manuscript:

HCC	Hepatocellular carcinoma
MAGE	Melanoma-associated antigen
TCGA	The Cancer Genome Atlas
MAFLD	Metabolic dysfunction-associated fatty liver disease
CAR-T	Chimeric antigen receptor T-cell
CTA	Cancer-testis antigen
AHE	Ammonium hydroxide enhancement
CC	Control chow
CCN	Control chow with AHE
HFC	High-fat casein
HFCN	High-fat casein with AHE
HFB	High-fat beef protein
HFBN	High-fat beef protein with AHE
Cyp51	Lanosterol 14α-demethylase
LKO	Hepatocyte-specific knockout
RT-qPCR	Reverse transcription quantitative PCR
RNA-seq	RNA sequencing
CDG	Cancer data gateway
DEA	Differential Expression Analysis
DEG	Differentially expressed gene
CI	Confidence interval
SD	Standard deviation
SEM	Standard error of the mean
HR	Hazard ratio
C-index	Concordance index
FDR	False discovery rate
