# Supplementary material for: Cross-Species Upregulation of MAGED2 in Liver Cancer Suggests a Role in Obesity-Driven Tumor Progression
Source: Curr Issues Mol Biol. 2026 Jan 27;48(2):139. doi: 10.3390/cimb48020139 (PMC12939545; doi:10.3390/cimb48020139)
Supplement: Supplementary file 1 [file cimb-48-00139-s001.zip › Sup-rev/Supplemental figures_revised.2.pdf]

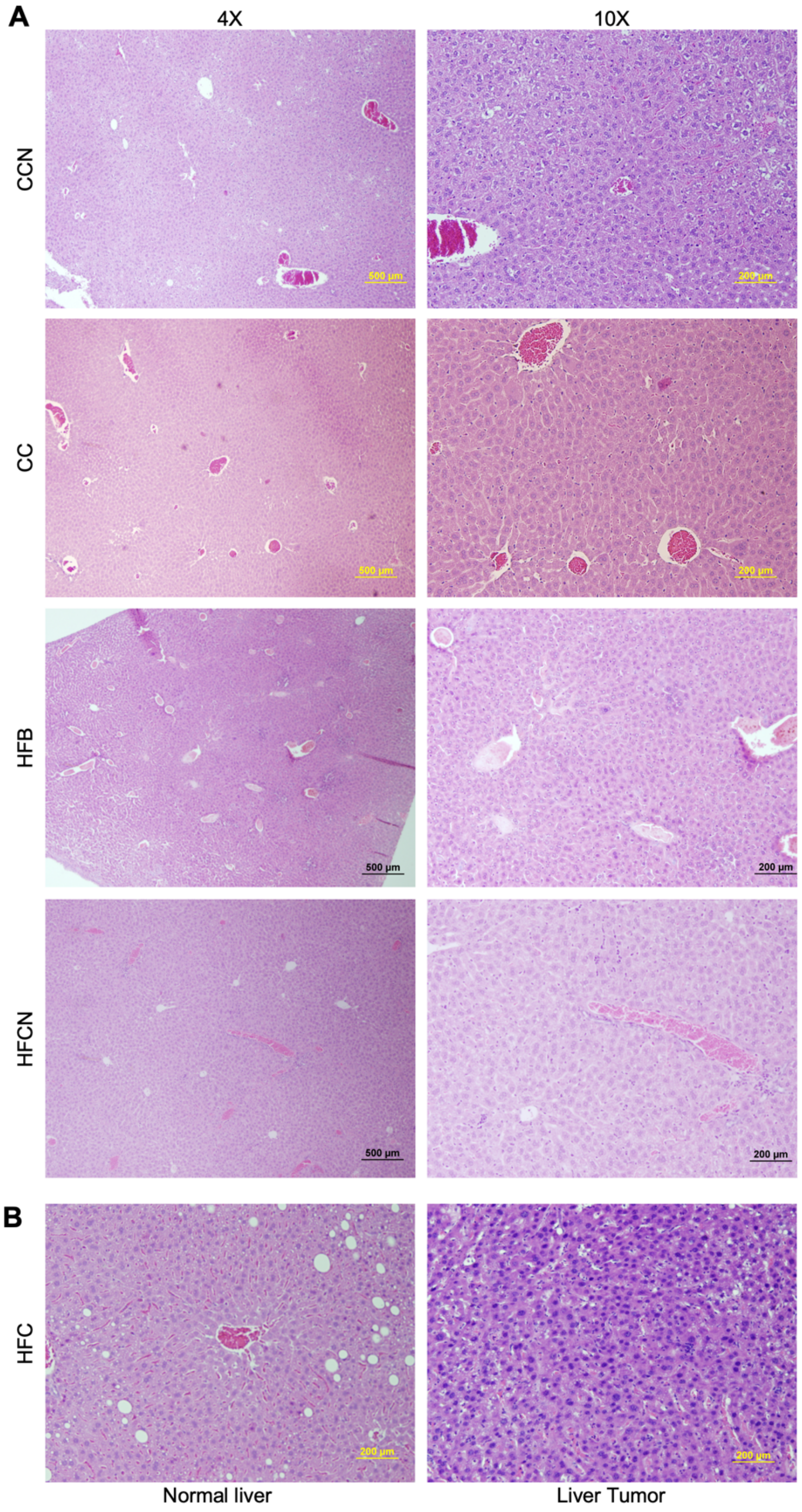

Figure S1. Liver tissue sections from 18-month-old male C3H/HeJ mice on different diets. (A) Tissue sections of normal livers from mice fed the indicated diets were stained with H&E and imaged at 4X (*left column*) and 10X magnification (*right column*). (B) Normal liver and tumor sections from the same mouse fed an HFC diet were imaged at 10X magnification.

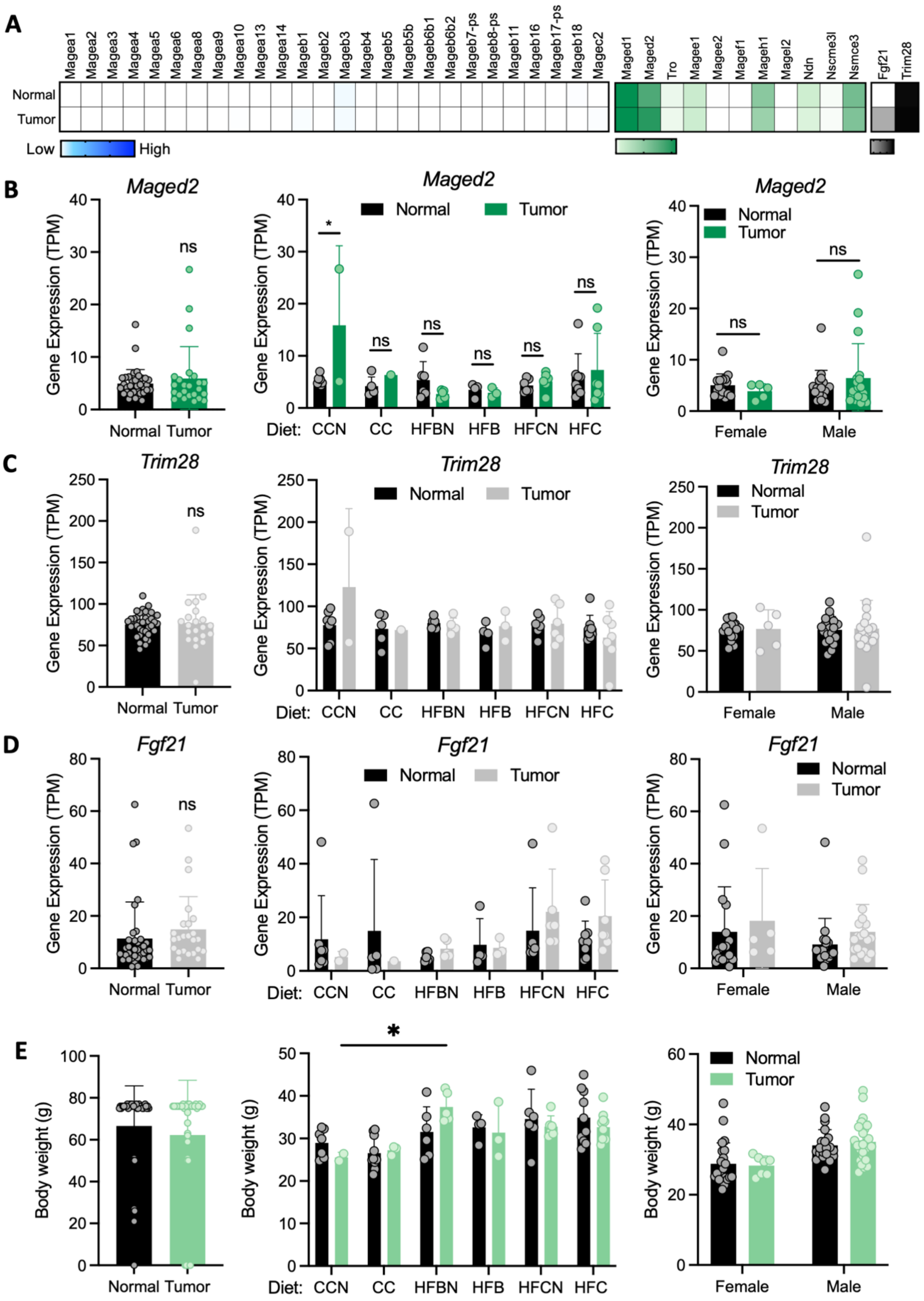

Figure S2. RNA-seq analysis of *Mage* expression in livers from mouse models of chronic diet-induced obesity. A) Heatmap shows the average TPM values for all normal liver and liver tumor samples from mice.

Expression levels of *Maged2* (B), *Trim28* (C), and *Fgf21* (D) as determined by RNA-seq for normal liver and liver tumor samples (*left graph*), divided by diet (*middle graph*) or gender (*right graph*). (E) *Body weight in grams at the time of tissue collection for animals with normal liver or liver tumors (left panel), stratified by diet (middle panel) or sex (right panel). Statistical significance was determined using an unpaired t test for comparisons of gene expression or body weights (E) between normal and tumor samples (left panel) and two-way ANOVA followed by Šídák's multiple-comparisons test for comparisons across diet groups or between sexes (middle and right panels)* [$P \leq 0.05$ (∗), $P \geq 0.05$ (non-significant, ns)].

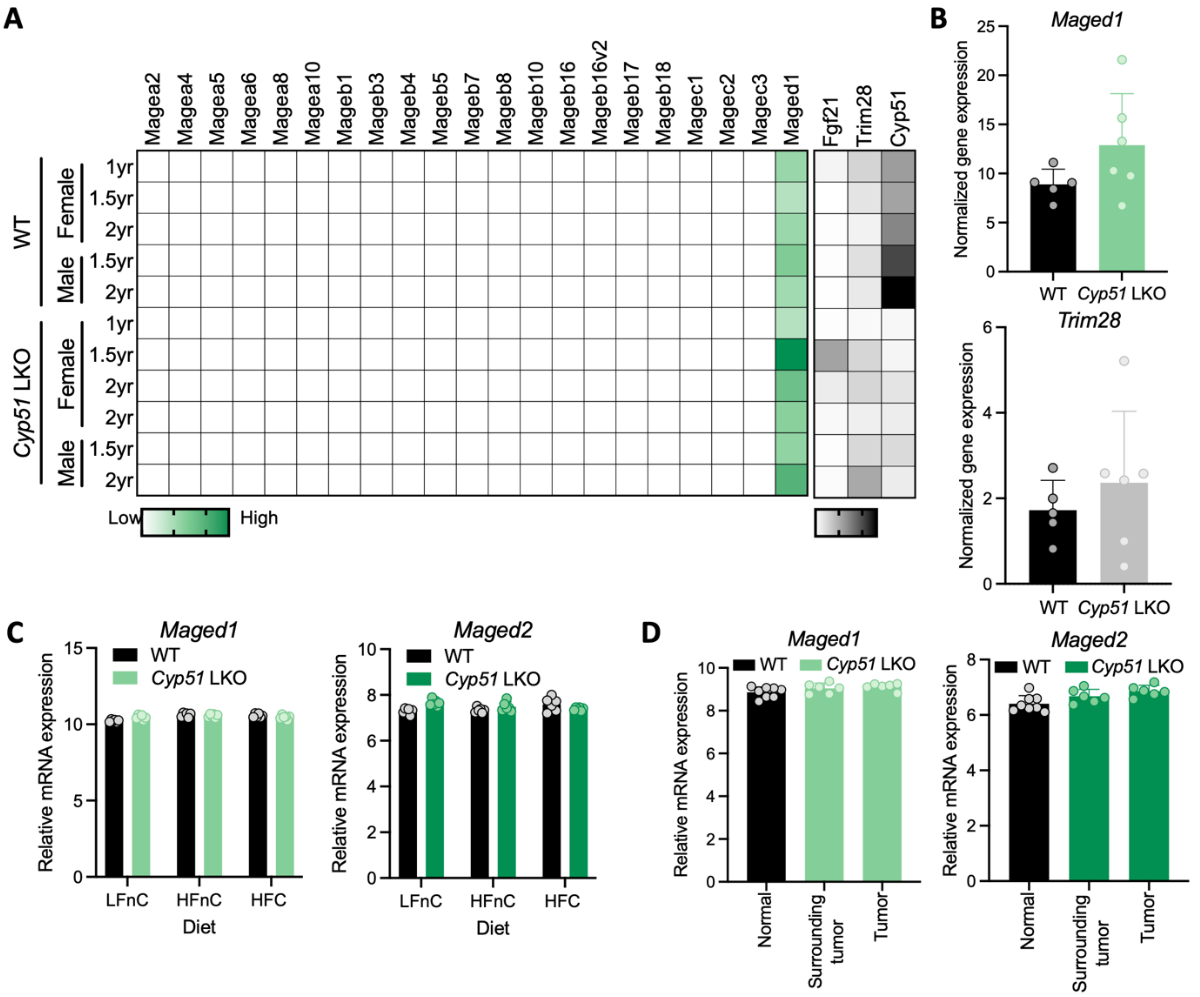

Figure S3. *Mage* expression in livers from *Cyp51* LKO mice. A) Heatmap shows the expression values, as determined by RT-qPCR, for spontaneous liver tumors from individual WT and *Cyp51* LKO mice. B) Graphs show expression levels of *Maged1* (*top*) and *Trim28* (*bottom*). C) Hepatic *Maged1* and *Maged2* expression levels in WT and *Cyp51* LKO mice fed either the standard laboratory diet without cholesterol (LFnC), high-fat diet without cholesterol (HFnC), or high-fat diet with cholesterol (HFC). Microarray dataset (GSE58271) was downloaded on March 18, 2025. D) Graphs show *Maged1* and *Maged2* expression levels in indicated liver tissues from WT and *Cyp51* LKO mice. Microarray dataset (GSE127772) was downloaded on March 18, 2025.

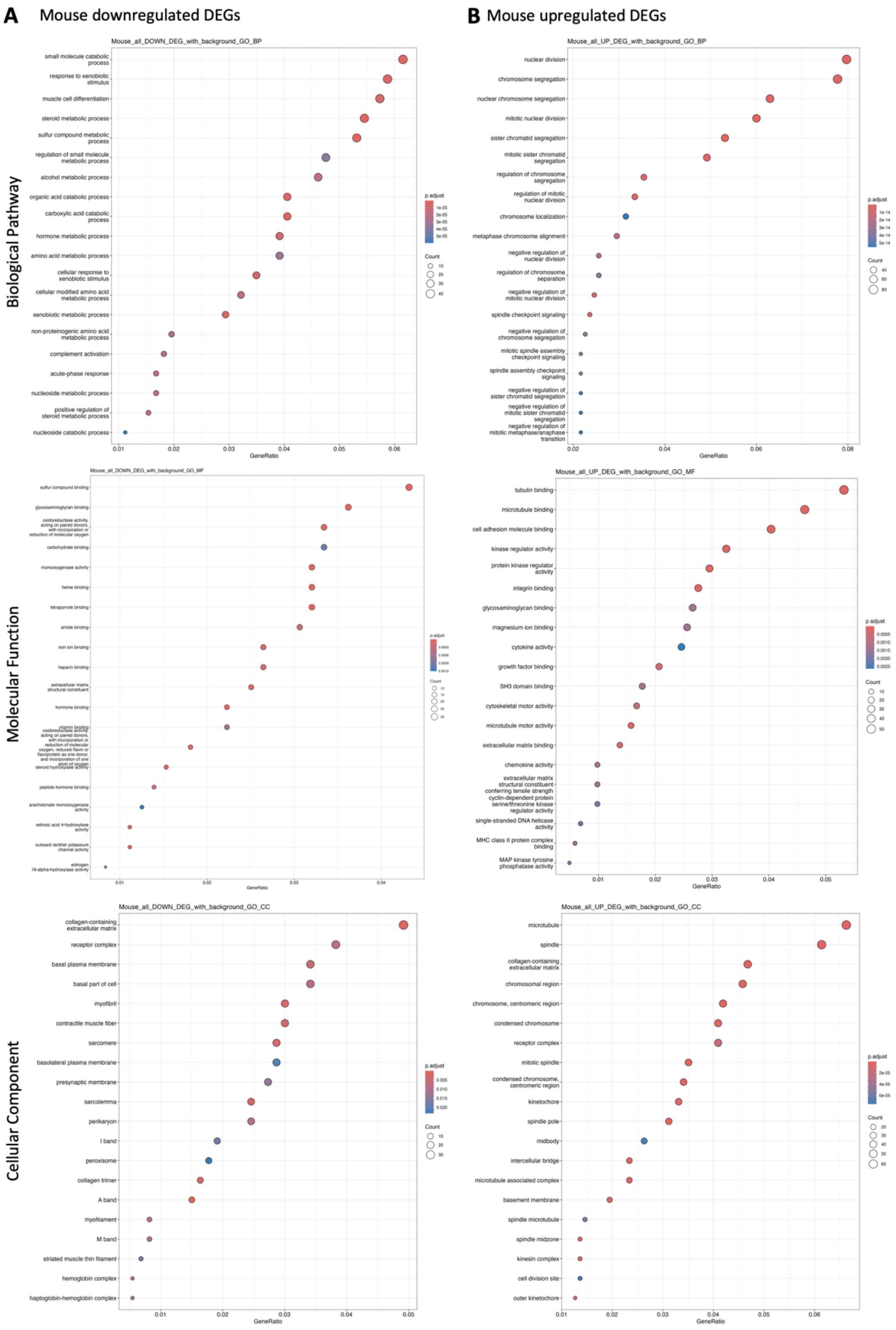

Figure S4. Gene ontology enrichment analyses of mouse DEGs. The results of the gene ontology enrichment analyses are shown for the (A) downregulated and (B) upregulated DEGs, with enrichment in biological pathway (*top*), molecular function (*middle*), and cellular component (*bottom*). clusterProfiler package in RStudio was used to perform these analyses and generate the dot plots.

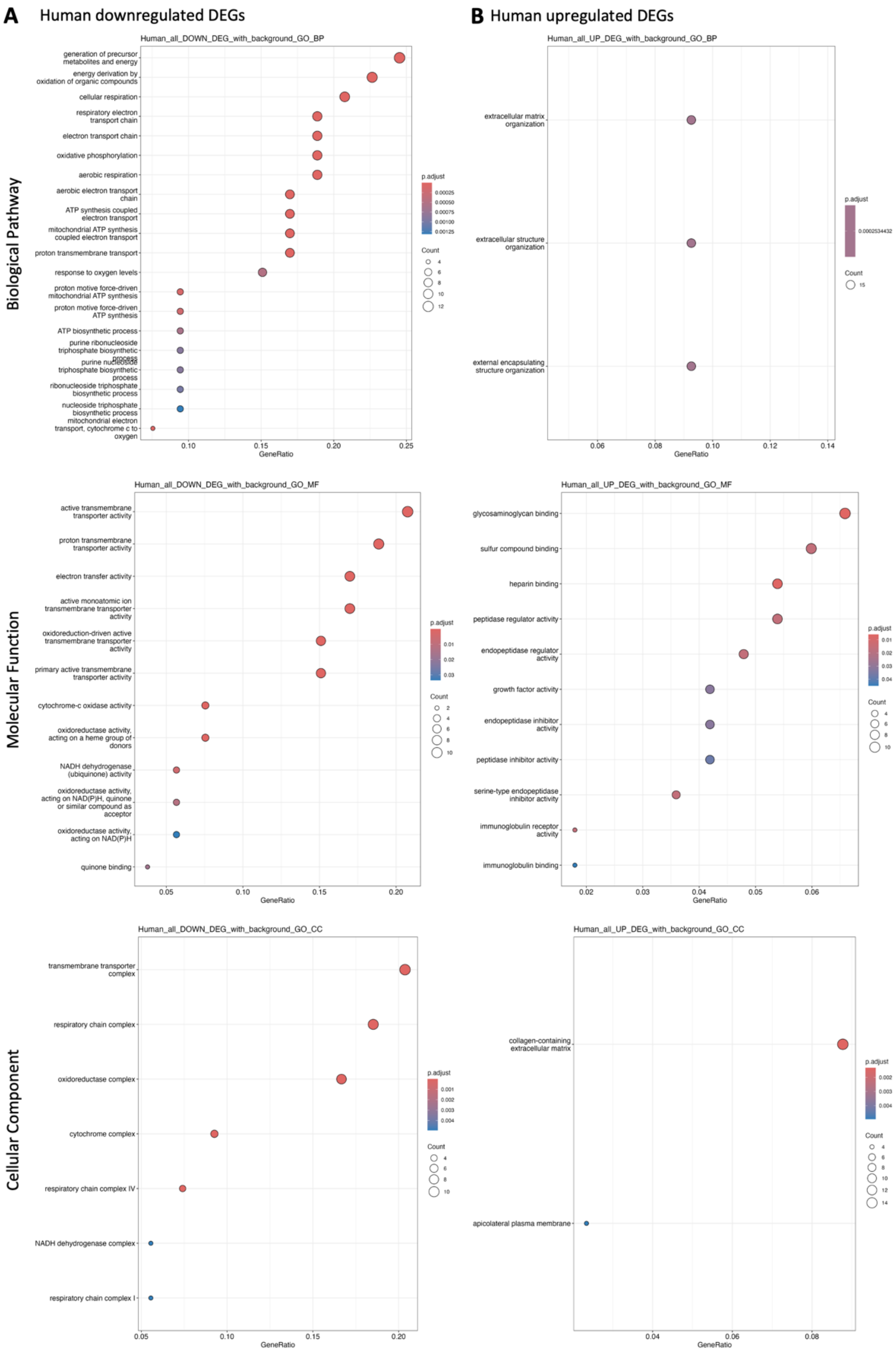

Figure S5. Gene ontology enrichment analyses of human DEGs. The results of the gene ontology enrichment analyses are shown for the (A) downregulated and (B) upregulated DEGs, with enrichment in biological pathway (*top*), molecular function (*middle*), and cellular component (*bottom*). clusterProfiler package in RStudio was used to perform these analyses and generate the dot plots.
